# CMEpiNet: Complex-Valued Multimodal Epilepsy Detection Network Model

**DOI:** 10.3390/s26134186

**Published:** 2026-07-02

**Authors:** Tianyi Su, Haiyan Zhu, Shuai Chen, Haifeng Wang

**Affiliations:** 1 Department of Electrical and Information Engineering, Shandong University of Science and Technology, Jinan 250031, China; sutianyi@sdust.edu.cn; 2 School of Physical Education and Health Linyi, Linyi University, Linyi 276012, China; zhuhaiyan@lyu.edu.cn; 3 School of Information Science and Engineering, Linyi University, Linyi 276012, China

**Keywords:** feature fusion, machine learning, deep learning, epilepsy detection, classification

## Abstract

Existing seizure detection methods cannot fully exploit the spatiotemporal features of multimodal signals. They also fail to capture deep associations among cross-modal features. This limits their ability to learn unified representations of spatiotemporal dependencies. This work proposes CMEpiNet (Complex-valued Multimodal Epilepsy detection Network model) to address this issue. CMEpiNet first uses complex-valued convolutions for feature extraction. It explicitly models phase synchronization, phase shifts, and cross-frequency coupling. Thus, EEG, ECG, and EMG features are represented in the complex-valued domain. During feature fusion, CMEpiNet uses a two-level semantic alignment-based fusion method. It applies cross-modal consistency constraints in a shared alignment space. It also performs distribution-level alignment in an epilepsy-related semantic latent space. These operations ensure the consistency of multimodal features in the global semantic structure. Finally, CMEpiNet uses a spatial attention-guided 3D convolutional classifier. The classifier jointly models the temporal, feature, and modality dimensions. Experimental results on the SeizeIT2 dataset show that CMEpiNet improves seizure detection sensitivity, reduces the false alarm rate, and maintains stable performance under perturbations.

## 1. Introduction

Epilepsy is a common chronic neurological disorder characterized by seizures that are inherently unpredictable [1]. Globally, approximately 30% of epilepsy patients experience uncontrolled seizures despite adherence to medication, and consequently face an elevated risk of sudden unexpected death (SUDEP) [2]. Electroencephalography (EEG) reflects the brain electrophysiological activity and has become a key clinical tool for observing seizures [3]. However, manual EEG interpretation is inherently subjective and dependent on scarce human expertise [4]. This manual process is not only labor-intensive but also error-prone; the need for prolonged review introduces fatigue, which fundamentally limits diagnostic accuracy and consistency. Therefore, AI-supported automatic epilepsy diagnosis has become an important solution [5].

Current automated seizure detection methods primarily rely on single-modality EEG signals. EEG signals mainly reflect cerebral electrical activity. Therefore, they provide limited information on interactions with other physiological changes, such as abnormal increases in heart rate and alterations in muscle activity. However, other physiological signals can contribute to early warning of epileptic seizures. Therefore, single-modality detection methods have clear limitations. Researchers use multimodal fusion to fully exploit physiological information from EEG, ECG, and EMG. Therefore, multimodal fusion-based seizure detection has become a new research trend. Multimodal signals can characterize epileptic seizure processes from multiple dimensions. These dimensions include neural electrical activity, cardiovascular responses, and muscle movements. They provide more comprehensive and complementary evidence for modeling complex physiological events. However, multimodal physiological signals differ significantly in noise characteristics, signal quality, and time–frequency structures. Under wearable acquisition conditions, they are susceptible to motion artifacts and poor electrode contact. Achieving robust multimodal feature fusion within a unified framework remains challenging, with two key issues remaining. (i) Existing studies often ignore deep oscillatory features related to phase and time-frequency structures in multimodal signals. This limits their ability to extract discriminative signal features. (ii) Cross-modal feature fusion lacks effective modeling of semantic consistency and distribution differences among modalities. This reduces model robustness under signal quality fluctuations and temporal asynchrony across modalities. To address the above issues, this work proposes CMEpiNet, a multimodal seizure detection model. CMEpiNet provides a multimodal feature optimization scheme that integrates complex-valued modeling, semantic alignment, and three-dimensional structural modeling. First, the complex-valued multimodal feature extraction module extracts time–domain features, phase features, and time–frequency structural features from analytic signals and complex-valued time–frequency spectra. This module fully uses the complementary information in amplitude, phase, and frequency-band energy distributions of multimodal signals. Second, the two-level semantic alignment fusion module performs cross-modal consistency constraints and shared latent space alignment. It reduces semantic and distributional biases among different modalities. Thus, the model obtains a more compact and consistent multimodal fusion representation. Finally, the classifier uses three-dimensional convolution and spatial attention. It jointly models the temporal, feature, and modality dimensions of the fused three-dimensional feature block. The main contributions are as follows.

(1) A complex-valued multimodal feature extraction method. This method uses complex-valued convolutions to explicitly model the coupling between signal amplitude and phase. It overcomes the limitation of traditional multimodal feature extraction methods that ignore phase information. It builds a dual-branch structure for time-domain-phase features and time-frequency features. In a unified complex-valued domain, it captures oscillatory features such as phase synchronization, phase shifts, and cross-frequency coupling. It unifies the representation forms of EEG, ECG, and EMG. It also extracts highly discriminative signal features.

(2) A two-level semantic alignment fusion method. This method constrains the multimodal fusion process from two aspects: representation-level consistency and distribution-level consistency. In the shared alignment space, it uses window-level soft matching and reliability gating to reduce semantic bias caused by temporal asynchrony and quality fluctuations across modalities. In the seizure-related semantic latent space, it aligns the global statistical structures of multimodal features. This reduces cross-modal distribution shifts. As a result, the model obtains stable cross-modal fusion representations under signal quality degradation and temporal asynchrony across modalities.

The rest of this paper is organized as follows. First, we introduce the multimodal seizure signal data and the corresponding preprocessing methods. Then, we describe the overall architecture and key module designs of the proposed complex-valued multimodal seizure detection model, CMEpiNet. Next, we evaluate the model through several comparative experiments and ablation studies. These experiments verify the effectiveness and robustness of each module in multimodal seizure detection. Finally, we present the conclusion and future work.

## 2. Related Work

Research on automated epilepsy detection has evolved from handcrafted rule-based pipelines to data-driven learning frameworks, driven largely by differences in feature construction, temporal modeling, and multimodal fusion mechanisms. Existing studies can be broadly grouped into four categories: statistical and nonlinear signal-based methods, classical machine-learning-based methods, deep-learning-based temporal and spatial modeling methods, and multimodal seizure detection methods.

(1) Statistical signal-based epilepsy detection methods

Early research on automatic EEG-based seizure detection was hindered by the challenge of objectively quantifying the complex and dynamic nature of brain signals. Conventional approaches operated in two stages: first, statistical properties were extracted from the time, frequency, or time–frequency domain; then, these were used to construct feature indicators with clear physical or statistical meaning. Seizure events were subsequently identified via thresholding or rule-based matching. Hjorth [6] introduced time-domain statistical parameters such as Activity, Mobility, and Complexity to characterize EEG energy level, temporal scale, and structural complexity, laying the foundation for subsequent statistical feature modeling. To address the limited spectral resolution of conventional Fourier analysis within short windows, Jansen et al. [7] systematically compared multiple autoregressive spectral estimation methods and found that AR modeling based on the Burg algorithm can yield higher-resolution spectral estimates under short-time-window settings. Gotman et al. [8] applied rhythmic statistical features to online seizure-trigger detection in long-term EEG and reduced missed detections by incorporating adaptive background comparison. Qu et al. [9] proposed patient-specific seizure-onset templates and a nearest-neighbor decision strategy to reduce false alarms.

Driven by a deeper understanding of EEG non-stationarity, research shifted from conventional time or frequency statistics toward time–frequency analysis, offering a more direct characterization of the transient structure of epileptic discharges. Adeli et al. [10] studied 3-Hz spike-and-wave complexes and systematically demonstrated that wavelet decomposition can localize transient spikes and slow-wave components simultaneously across multiple scales. This provides a tool better matched to multi-scale time–frequency representation of epileptic activity than Fourier spectra. Building on the time–frequency representation paradigm, researchers began using wavelet subband energies and related statistics as detection features and feeding them into learning-based classifiers. For example, Subasi [11] input DWT subband features into a neural network to improve detection robustness, representing a typical two-stage workflow of time–frequency feature extraction followed by statistical learning. Faust et al. summarized the broad use of wavelet methods in denoising, feature extraction, and classification, and observed that DWT—particularly db4—is the most commonly adopted approach in epilepsy EEG studies [12].

Second-order statistics are often insufficient for epileptic EEG. Non-Gaussianity is not fully captured. Nonlinear interactions are also missed. Later studies therefore incorporated complexity measures and higher-order statistics. Discriminative information is then enhanced. Kannathal et al. [13] systematically compared spectral entropy and embedding entropy and reported that seizure EEG tends to exhibit reduced complexity, while different entropy measures vary significantly in discriminative power. Chua et al. [14] further used bispectrum magnitude and its entropy-based features to characterize nonlinear phase coupling, improving discrimination among normal, pre-ictal, and ictal states within GMM/SVM frameworks. Acharya et al. [15] combined embedding entropy (ApEn, SampEn) with bispectrum-based phase entropy to perform three-class classification under a multi-classifier setting, validating the effectiveness of low-dimensional nonlinear features. Beyond entropy and higher-order spectral features, another line of work has explicitly investigated the nonlinear and sequential dynamics of epileptic activity. Yang et al. [16] used recurrence plots and recurrence quantification analysis on stereo-electroencephalography (SEEG) recordings to characterize dynamical differences among interictal, preictal, and ictal stages, as well as between epileptogenic and non-epileptogenic regions. Their results showed that epileptogenic channels exhibited more deterministic and recurrent structures, and that synchronization between epileptogenic channels was strengthened during seizures. Farahi et al. [17] further used recurrence-based nonlinear analysis to identify seizure dynamic trajectories from EEG recordings and to trace the sequence of regions involved during seizure evolution. These studies demonstrate that epileptic seizures exhibit rich nonlinear and sequential dynamical structures. However, they mainly rely on handcrafted recurrence-based descriptors and dynamical interpretation, rather than end-to-end multimodal representation learning for seizure detection.

Overall, handcrafted statistical and nonlinear-dynamical methods provide interpretable descriptions of epileptic EEG, including spectral changes, complexity variations, phase coupling, recurrence structures, and seizure-state transitions. However, these methods usually depend on predefined descriptors and fixed analysis assumptions. The extracted features are not jointly optimized with the downstream detection objective, and most studies focus on EEG or SEEG alone. Therefore, although they provide important evidence that seizures are dynamic processes, their integration with multimodal deep representation learning remains limited.

(2) Classical machine-learning-based epilepsy detection methods

Building on statistical signal processing, researchers gradually combined handcrafted EEG features with classical machine learning models. This strategy reduces the instability introduced by manually tuned thresholds and improves the consistency and automation of detection decisions. Such methods typically follow a two-stage pipeline. First, signal processing and handcrafted rules are used to construct a fixed feature representation. Second, a supervised learning model is trained to discriminate seizure states. In these approaches, learning mainly operates at the decision layer, while the feature representation itself is not updated toward the task objective.

In terms of feature construction, researchers expanded the feature families developed in the statistical signal-processing stage. Polat et al. [18] combined FFT-based frequency-domain features with a decision-tree classifier to distinguish epileptic EEG from normal EEG, demonstrating the feasibility of conventional spectral features in a supervised learning setting. However, spectral features implicitly assume signal stationarity and thus have limited ability to characterize transient changes at seizure onset. To better capture time-varying frequency components during seizures, Tzallas et al. [19] extracted local energy-distribution features from time–frequency representations to describe evolving patterns on the time–frequency plane, achieving better detection performance than purely frequency-domain analysis. Because second-order statistics are often insufficient to represent complex dynamics, distribution-based and nonlinear feature representations were also introduced. Orhan et al. [20] clustered wavelet coefficients and computed distributional statistics to better describe the global shape of coefficient patterns. Chua et al. [21] extracted higher order spectral (HOS) features from EEG signals to characterize their nonlinear and non-Gaussian properties, achieving class-specific feature ranges with high statistical significance (*p* = 0.002). Nicolaou and Georgiou [22] employed permutation entropy as a low-complexity feature for automated epileptic seizure detection using an SVM classifier.

In classifier design and system optimization, related research has gradually shifted away from single discriminative models. Increased emphasis has been placed on system stability and engineering practicality. Subasi [23] proposed a seizure EEG classification framework based on discrete wavelet transform and a Mixture of Experts model. A gating mechanism coordinates multiple expert models. It adapts to different feature distributions. Sensitivity to feature heterogeneity is reduced. This limitation is common in single classifiers. To improve performance while retaining interpretability, Kabir et al. [24] introduced Logistic Model Trees, combining tree structures with logistic regression to obtain smoother decision boundaries. Mursalin et al. [25] evaluated multiple feature-classifier combinations from a system perspective and used feature-selection strategies to reduce the impact of redundant features on classification performance.

Overall, classical machine-learning-based epilepsy detection introduces supervised discrimination on top of statistical features, enabling a transition from heuristic thresholds to data-driven decisions. These methods still follow a modeling paradigm that assumes predefined feature representations. Feature construction is separated from classification decisions. Joint optimization of the feature representation is not possible during learning. This structural limitation makes it difficult to fully characterize the continuously evolving dynamics of seizures and leads to clear weaknesses in cross-patient generalization and adaptability to complex clinical scenarios.

(3) Deep-learning-based epilepsy detection methods

To overcome the limitations of handcrafted features in representational power and generalization, researchers have increasingly shifted toward deep-learning-driven modeling. The key idea of these methods is to jointly optimize representation learning and discriminative decision-making. Models can automatically learn multi-scale and high-dimensional discriminative representations from raw or minimally preprocessed EEG signals. Uncertainties caused by signal non-stationarity, inter-subject variability, and complex artifacts are mitigated to some extent.

For automatic feature representation and spatial-structure extraction, convolutional neural networks (CNNs) have become a widely used choice due to their strength in capturing local patterns. Thodoroff et al. [26] addressed the heavy reliance on handcrafted features in traditional methods and their insufficient robustness under complex background EEG by using deep networks to learn discriminative features directly from EEG, thereby reducing dependence on feature engineering. Acharya et al. [27] demonstrated that a deep CNN could classify seizure EEG signals without manual feature extraction, showing the potential of end-to-end learning for automated seizure detection. To better align with clinical visual inspection, Emami et al. [28] demonstrated that CNN applied to raw EEG plot images could detect seizures in a manner analogous to the visual inspection performed by clinical epileptologists. For real-time applications, Daoud and Bayoumi [29] emphasized deployability by using lightweight architectures and channel selection to reduce inference cost. However, CNNs are fundamentally built on local receptive fields and are better at capturing local morphology or texture-like features, while they are relatively less effective at modeling long-range evolution across time scales. Although image-based representations and lightweight designs improve usability, they can make models more sensitive to preprocessing strategies and capacity constraints, thereby reducing stability across scenarios.

To better exploit temporal dynamics, recurrent neural networks (RNNs) and their variants have been used to model EEG time evolution. Hussein et al. [30] employed a deep LSTM network to model temporal dependencies in EEG signals, enabling robust seizure detection under both ideal and artifact-contaminated conditions. As the research focus expanded from single-channel sequences to inter-channel interactions, brain-network-structured features began to attract increasing attention. Mirowski et al. [31] highlighted the importance of cross-channel synchrony and dependency patterns in pre-seizure state discrimination, providing motivation for subsequent spatial-dependency modeling. Tang et al. [32] argued that conventional convolutions are limited in expressing non-Euclidean relationships and long-range spatial dependencies among channels and proposed graph neural networks (GNNs) to model electrode relationships, together with self-supervised learning to alleviate insufficient annotations. Li et al. [33] addressed the difficulty of jointly modeling spatiotemporal dependencies, spectral hierarchical structure, and uncertainty in the pre-seizure interval by proposing spatiotemporal-frequency hierarchical graph convolution combined with semi-supervised active learning, and further introduced an adaptive mechanism for estimating patient-specific pre-seizure intervals to reduce label noise. Nevertheless, graph-based methods are highly dependent on graph construction strategies and prior assumptions, and dynamic or hierarchical graphs markedly increase computational complexity, which makes cross-center transfer and real-time deployment challenging.

(4) Multimodal seizure detection methods

Multimodal physiological sensing has also become an important direction for seizure detection, especially in wearable and long-term monitoring scenarios. Chen et al. [34] reviewed seizure detection studies using portable and wearable multimodal signals, including EEG, ECG, EMG, accelerometry, electrodermal activity, and other non-cerebral physiological signals. Their review showed that multimodal systems can provide complementary information for ambulatory seizure monitoring. Yang et al. [35] proposed a multimodal artificial intelligence system combining EEG and ECG for seizure identification, and demonstrated improved out-of-distribution generalization across clinical datasets. Zhang et al. [36] evaluated wearable EEG, EMG, and accelerometry for tonic-clonic seizure detection using data from the SeizeIT2 study, and showed that combining wearable EEG with extracerebral modalities can substantially reduce false positive rates while retaining high sensitivity. These studies indicate that multimodal fusion is not limited to simple feature concatenation and has already been explored in epilepsy detection. Nevertheless, many existing multimodal approaches are designed for specific seizure types, modality combinations, or decision-level fusion settings. The joint modeling of phase-aware time–frequency dynamics, cross-modal temporal asynchrony, modality reliability, and high-level semantic consistency remains insufficiently explored.

Although deep learning has advanced epilepsy detection from fixed feature representations to jointly learned representations and has greatly improved automation, it still faces several challenges. These challenges include substantial dependence on labeled data. Stability is reduced by ambiguous boundaries of the pre-seizure interval. Generalization is degraded by distribution shifts across patients and medical centers. Overall, despite substantial progress from rule-based approaches to machine learning and deep learning, existing epilepsy detection methods still suffer from the following limitations: (1) Many EEG-based studies focus mainly on amplitude variations in the time or frequency domain. Temporal dependencies between signals are often overlooked. Sequential dynamical characteristics are not extracted sufficiently. The ability to characterize and recognize early pathological changes is therefore weakened. (2) Existing multimodal fusion methods often rely on simple feature concatenation and lack effective modeling and alignment of semantic consistency across modalities during representation learning. As a result, cross-modal complementarity is not fully exploited. Redundant information is not effectively suppressed. Multimodal epilepsy detection models are therefore obtained with limited robustness and weak generalization.

## 3. Materials and Methods

### 3.1. Model Architecture

**Figure 1:**
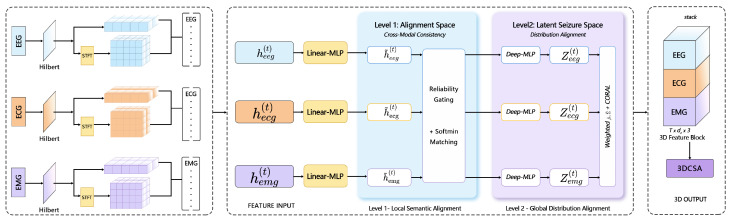
Overall framework of CMEpiNet. EEG, ECG, and EMG signals are first encoded by the shared complex-valued feature extractor (CMFE), then aligned across modalities by the dual-level semantic alignment fusion module (DLSAF). The aligned features are stacked into a T×d×3 feature block and classified as seizure or non-seizure by the 3D spatial-attention classifier (3DCSA).

EEG, ECG, and EMG in multimodal epilepsy detection differ substantially in sampling characteristics, noise levels, and representation forms. As a result, it is difficult to align and fuse multimodal information within a unified feature space. This section introduces the multimodal detection model CMEpiNet (Complex-valued Multimodal Epilepsy Network), as shown in Figure 1. The model consists of a Complex-valued Multimodal Feature Extractor (CMFE), a Dual-Level Semantic Alignment Fusion module (DLSAF), and a 3D convolutional neural network with a spatial attention mechanism (3DCSA). The main working mechanism of CMEpiNet is described as follows:

(1) Feature extraction module. We adopt a unified Complex-valued Multimodal Feature Extractor (CMFE) for all modalities. For each modality, CMFE builds an isomorphic dual-branch complex-valued convolutional network. The time-domain-phase branch takes the analytic signal as input and uses 1D complex-valued convolutions to extract sequential oscillatory features from the joint amplitude-phase perspective. The time–frequency branch takes the complex-valued time–frequency spectrum as input and applies 2D complex-valued convolutions to capture band-energy distributions and cross-band coupling patterns. The complex-domain features produced by the two branches are then fused along the feature dimension, forming a modality-level representation that contains both time-domain-phase information and explicit frequency-domain information.

(2) Feature fusion module. Under constraints of cross-modal consistency and shared latent-space alignment, this module models a unified representation of multimodal features. First, the CMFE features of each modality are mapped into an alignment space with a unified dimensionality. For each time segment, a cross-modal consistency constraint is imposed on the EEG, ECG, and EMG features, so that multimodal representations originating from the same epileptic physiological event remain semantically consistent in the feature space. Next, the aligned modality features are projected by their respective projection networks into a shared latent seizure space, and a distribution-level alignment loss is used to reduce the global distribution discrepancies among modalities within this latent space. Finally, the latent representations of the three modalities are stacked along the modality dimension to form a 3D fused feature block that jointly includes the time, feature, and modality dimensions. This approach fully leverages the complementary information across modalities in amplitude-phase structure and time–frequency characteristics.

(3) 3DCSA classification module. A 3D convolutional neural network with an embedded spatial attention mechanism is used to deeply process the 3D fused feature blocks in the shared latent space. The feature block obtained by stacking the latent representations of EEG, ECG, and EMG is denoted as $F\in R^{T\times d_{z}\times n}$, where the three axes correspond to the time, feature, and modality dimensions. Using 3D convolutions, the network jointly models these three dimensions and progressively extracts both local and global structures of multimodal epileptic patterns. A spatial attention mechanism is then introduced to adaptively learn importance weights for each spatial location in the 3D convolutional feature maps, i.e., different time segments, latent feature channels, and modality combinations. Regions that are more relevant to seizure-state discrimination receive higher responses. Noise and redundant information are suppressed. Finally, the weighted high-dimensional features are flattened and fed into a fully connected layer and a Softmax classifier to output the class probabilities of different epilepsy states.

### 3.2. Data Preprocessing

To construct input samples suitable for model training, this work applies a unified preprocessing procedure to the SeizeIT2 dataset. The model is trained on the training subset, which includes subjects sub-001 to sub-096. The recordings consist of dual-channel EEG, single-channel ECG, and single-channel EMG. All signals were sampled at 256 Hz. First, signals from the three modalities are filtered. A 0.5–60 Hz band-pass filter is applied to remove DC drift and high-frequency noise. A 49.5–50.5 Hz notch filter is applied to suppress power-line interference. Then, segments labeled as impaired signal quality (IMPD) are excluded from the dataset. These segments are not used to construct training samples. This helps ensure the validity of the training data. A sliding-window strategy is used to segment the continuous signals. The window length is set to 2 s, corresponding to 512 samples. For seizure segments, dense sliding windows with a step size of 0.5 s are used to cover the onset and offset stages of seizures. For background segments, sliding windows with a step size of 1 s are used. The segmented windows are further organized into temporal sequences. Each input sequence contains T=30 consecutive windows. For seizure sequences, a step size of 5 windows is used for sequence sampling. For background sequences, a step size of 15 windows is used. If a time gap longer than 3 s exists between adjacent windows, the sequence is split at the gap. The resulting parts are then treated as independent sequences. This process avoids interference from cross-gap sequences during temporal modeling. Class balance is then handled for the training samples. Background sequences are uniformly sampled across patients. The total number of background sequences is controlled at five times the number of seizure sequences. This alleviates the class imbalance between seizure and background samples. The final training samples are randomly shuffled at the patient level. Finally, window-level signal normalization is performed. For each window, z-score normalization is applied to each channel separately. This process reduces amplitude differences across subjects, modalities, and channels.

Through the above preprocessing procedure, training samples with stable signal quality, relatively balanced classes, and suitability for multimodal temporal modeling are obtained.

### 3.3. Complex-Valued Multimodal Feature Extractor (CMFE)

Multimodal seizure-related signals show clear heterogeneity in noise statistics and signal structures. Features based only on amplitude or spectral distributions cannot effectively characterize seizure-related changes across different modalities within a unified framework. Existing studies have shown that abnormal changes can be observed before seizure onset. These changes appear in inter-regional phase relationships. They also appear in the temporal organization of neural activity. Such changes occur earlier than prominent discharge events. These findings indicate that phase information plays an important role in seizure prediction. In addition, frequency-domain and time–frequency features complement the temporal information reflected by phase by describing energy organization and dynamic evolution, thereby enabling a more comprehensive representation of the pre-seizure state.

Existing seizure detection methods cannot fully exploit deep oscillatory features in multimodal signals. These features include phase characteristics, time-frequency structures, temporal dependencies, and spatially coordinated changes. This limits the model’s ability to accurately represent complex spatiotemporal dynamics during seizures. To address this limitation, this section proposes a complex-valued multimodal feature extraction module, termed the Complex-valued Multimodal Feature Extractor (CMFE), which consists of a time-domain-phase branch and a time–frequency branch. Both branches are centered on complex-valued convolutions. By jointly processing the real and imaginary parts in the complex domain, the model can preserve phase variations as well as frequency- and amplitude-related information during feature extraction, thereby providing a more complete description of seizure-related dynamics in multimodal signals. This module also helps establish a consistent feature description across modalities, laying the foundation for joint modeling of seizure-related patterns.

CMFE adopts a parallel processing architecture to deeply fuse phase and amplitude-structure information in the complex domain. For each modality, CMFE includes two parallel complex-valued convolutional pathways. The time-domain-phase branch takes the analytic signal as input and focuses on capturing the dynamic characteristics of amplitude and instantaneous phase from the time series. The time–frequency branch takes the complex-valued time–frequency spectrum as input and focuses on extracting band-structure information and cross-band coupling features from the time–frequency plane. Both pathways perform convolution in the complex domain, with one emphasizing sequential phase-related features and the other emphasizing explicit frequency-domain structural features. Their outputs are then efficiently fused along the feature dimension, producing a unified complex-domain feature vector for the modality.

**Figure 2:**
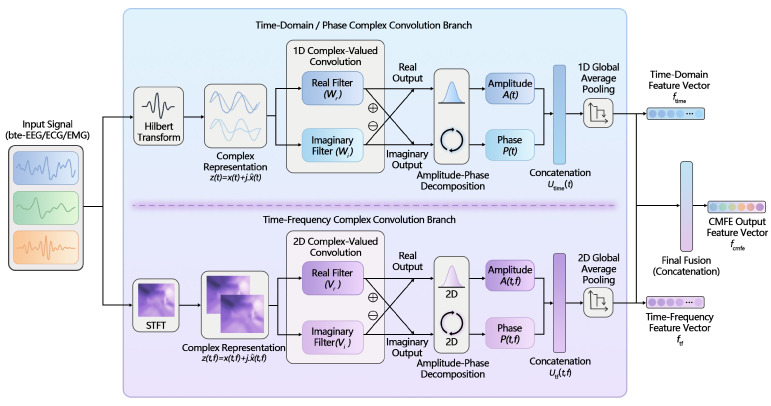
Architecture of the CMFE module. For each modality, the analytic signal is processed by two parallel complex-valued branches: a 1D time-domain phase branch and a 2D STFT-based time-frequency branch. The resulting amplitude and phase features are pooled and concatenated to form the complex-domain feature vector for that modality.

As shown in Figure 2, CMFE processes each modality’s input time series with two parallel branches—a time-domain/phase complex convolution branch and a time–frequency complex convolution branch, to generate a time-domain feature vector and a time–frequency feature vector, respectively. These vectors are concatenated along the feature dimension to form the CMFE output vector $f_{cmfe}$.

**(1)** 
**Time-domain-phase complex convolution branch**

As shown in the upper part of Figure 2, this branch takes each preprocessed single-channel time series (EEG, ECG, EMG) as input. A Hilbert transform is first applied to construct the corresponding analytic signal:

(1) $$x_{a}\left(t\right)=x\left(t\right)+iH\left[x\left(t\right)\right],$$

where x(t) is the original continuous-time signal and H[x(t)] denotes its Hilbert transform. In practical implementations, the signal is discretely sampled at the sampling rate $F_{s}$. The physical time corresponding to the *n*-th sample is

(2) $$t_{n}=\frac{n}{F_{s}},\;n=0,1,\ldots ,N-1.$$

Accordingly, the discrete analytic signal $x_{a}\left[n\right]$ is defined as the value of $x_{a}\left(t\right)$ at the sampling instants $t_{n}$, i.e.,

(3) $$x_{a}\left[n\right]=x\left[n\right]+iH\left\{x\left[n\right]\right\}.$$

After constructing the analytic signal via the Hilbert transform, x[n] serves as the real part and H{x[n]} serves as the imaginary part. This unified complex representation preserves both amplitude and phase information.

As shown in the middle of the upper part of Figure 2, the analytic signal $x_{a}\left[n\right]$ is then fed into a 1D complex-valued convolutional network. The network is composed of three cascaded 1D complex convolution layers, which progressively model oscillatory frequency characteristics and phase variation patterns within a local temporal range.

The complex-valued convolution kernel of the *l*-th layer is denoted as

(4) $$W^{\left(l\right)}=W_{r}^{\left(l\right)}+iW_{i}^{\left(l\right)},\;l\in \left\{1,2,3\right\}.$$

Here, *l* is the layer index, and $W_{r}^{\left(l\right)}$ and $W_{i}^{\left(l\right)}$ represent the learnable real and imaginary parameters of the *l*-th complex kernel, respectively.

To enable each kernel to simultaneously select frequency components and model phase variations within a local temporal window, we parameterize the 1D complex kernel of each layer as a Gaussian-windowed complex exponential:

(5) $$w_{k}^{\left(l\right)}\left(t\right)=\exp\;\left(-\frac{t^{2}}{2{\left(\sigma_{k}^{\left(l\right)}\right)}^{2}}\right)\exp\;\left(i\;\left(2\pi f_{k}^{\left(l\right)}t+\phi_{k}^{\left(l\right)}\right)\right).$$

Here, *k* is the kernel index; $f_{k}^{\left(l\right)}$ is the center frequency of the *k*-th kernel in layer *l*; $\sigma_{k}^{\left(l\right)}$ controls the temporal support of the Gaussian window; and $\phi_{k}^{\left(l\right)}$ is the phase offset parameter. In the discrete implementation, we set $t=n/F_{s}$, discretize the kernel, and use it for 1D discrete complex convolution. After expansion, the real and imaginary parts are:

(6) $$w_{k,r}^{\left(l\right)}\left[n\right]=\exp\;\left(-\frac{{\left(n/F_{s}\right)}^{2}}{2{\left(\sigma_{k}^{\left(l\right)}\right)}^{2}}\right)\cos\;\left(2\pi f_{k}^{\left(l\right)}\frac{n}{F_{s}}+\phi_{k}^{\left(l\right)}\right),$$

(7) $$w_{k,i}^{\left(l\right)}\left[n\right]=\exp\;\left(-\frac{{\left(n/F_{s}\right)}^{2}}{2{\left(\sigma_{k}^{\left(l\right)}\right)}^{2}}\right)\sin\;\left(2\pi f_{k}^{\left(l\right)}\frac{n}{F_{s}}+\phi_{k}^{\left(l\right)}\right).$$

With this parameterization, EEG, ECG, and EMG can be modeled using a unified convolutional kernel form.

The forward propagation of the three complex convolution layers can be written recursively as

(8) $$Z^{\left(1\right)}=X*W^{\left(1\right)},\;Z^{\left(2\right)}=Z^{\left(1\right)}*W^{\left(2\right)},\;Z^{\left(3\right)}=Z^{\left(2\right)}*W^{\left(3\right)},$$

where ∗ denotes 1D complex convolution, $X=x_{a}\left[n\right]$ is the input analytic signal sequence, and $Z^{\left(l\right)}$ is the feature map produced by the *l*-th layer. The third-layer output $Z^{\left(3\right)}$ is a complex-valued feature map, whose real and imaginary parts are denoted by $Z_{r}^{\left(3\right)}$ and $Z_{i}^{\left(3\right)}$, respectively.

In the complex-valued feature map, the real part mainly characterizes amplitude-related features such as response strength within a local temporal range, energy distribution, and waveform shape. The imaginary part is more sensitive to changes in relative timing relationships across time points, and it can be used to describe phase variations, phase discontinuities, and changes in synchronization relationships.

To further reveal the amplitude and phase information contained in the third-layer complex feature map, as shown on the right side of the upper part of Figure 2, we perform amplitude–phase decomposition only on $Z^{\left(3\right)}$ to obtain the amplitude and phase components:

(9) $$A\left(t\right)=\left|Z^{\left(3\right)}\left(t\right)\right|,\;P\left(t\right)=∠Z^{\left(3\right)}\left(t\right).$$

Corresponding to “Amplitude A(t)” and “Phase P(t)” in the figure, the amplitude and phase can be computed from the real and imaginary parts of the third-layer output:

(10) $$A\left(t\right)=\sqrt{{\left(Z_{r}^{\left(3\right)}\left(t\right)\right)}^{2}+{\left(Z_{i}^{\left(3\right)}\left(t\right)\right)}^{2}},\;P\left(t\right)=\mathrm{atan}2\;\left(Z_{i}^{\left(3\right)}\left(t\right),\;Z_{r}^{\left(3\right)}\left(t\right)\right).$$

Here, atan2(·,·) is the two-argument arctangent function with quadrant correction.

Amplitude–phase decomposition is applied only to the third-layer output in order to preserve the representational capacity of complex convolution layers while avoiding unnecessary nonlinear decomposition in shallow layers.

The amplitude and phase components are then concatenated along the channel dimension to obtain a joint time-domain representation:

(11) $$U_{\mathrm{time}}\left(t\right)=\left[A\left(t\right),P\left(t\right)\right].$$

Here, *T* is the number of time steps within the segment, and [·,·] denotes concatenation along the channel dimension.

Next, 1D global average pooling (GAP) is applied to $U_{\mathrm{time}}\left(t\right)$, i.e., a global average over the time dimension is computed for each channel, yielding a fixed-length time-domain feature vector:

(12) $$f_{\mathrm{time}}={\mathrm{GAP}}_{1D}\left(U_{\mathrm{time}}\right),\;f_{c}=\frac{1}{T}\sum_{n=1}^{T}U_{\mathrm{time}}\left(c,n\right).$$

Here, $U_{\mathrm{time}}\left(c,n\right)$ denotes the response at the *c*-th channel and the *n*-th time step in the joint representation. This operation aggregates amplitude and phase statistics over the entire segment along the time dimension.

Finally, the time-domain feature vector $f_{\mathrm{time}}$ is passed through a linear transformation and then normalized to obtain the time-domain–phase embedding vector for the segment:

(13) $$h_{t}=W_{t}f_{\mathrm{time}}+b_{t}.$$

Here, $W_{t}$ and $b_{t}$ are learnable parameters. The resulting $h_{t}$ serves as the time-domain–phase feature vector of the current modality for this segment and is used for final fusion with the output of the other branch (the time–frequency branch).

**(2)** 
**Time–frequency complex convolution branch and feature vector generation**

As shown in the lower part of Figure 2, the time–frequency complex convolution branch takes the discrete analytic signal $x_{a}\left[n\right]$ (output from the time-domain–phase branch) as input. It first applies the short-time Fourier transform (STFT) to map the 1D complex sequence to a 2D complex-valued time–frequency map X(f,τ), thereby introducing structural information in the frequency and time–frequency domains. In the continuous-time domain, STFT is defined as

(14) $$Z\left(f,\tau \right)=\int_{-\infty }^{+\infty }x_{a}\left(t\right)\;w\left(t-\tau \right)\;e^{-i2\pi ft}\;dt,$$

where w(·) is the window function, *f* is the frequency variable, and τ is the time parameter corresponding to the window position (or window center). X(f,τ)∈C denotes the complex coefficient at the time–frequency point (f,τ).

In practice, STFT is implemented in discrete time. Let $x_{a}\left[n\right]$ be the discrete analytic signal, with window length *L*, hop size *H*, and FFT size $N_{\mathrm{fft}}$. The discrete form can be written as

(15) $$Z\left(f,\tau \right)=\sum_{n=0}^{L-1}x_{a}\left[n+\tau H\right]\;w\left[n\right]\;e^{-i2\pi fn/N_{\mathrm{fft}}},\;f=0,\ldots ,F-1.$$

Here, *f* is the index of the discrete frequency bin, and τ is the time-frame index corresponding to the sliding STFT window along the time axis. After computing STFT for an input segment, we obtain a complex-valued time–frequency map $Z\in C^{F\times T}$. Decomposing it into real and imaginary parts yields

(16) $$Z=Z_{r}+iZ_{i},\;Z_{r}=ℜ\left(Z\right)\in R^{F\times T},\;Z_{i}=ℑ\left(Z\right)\in R^{F\times T}.$$

That is, the STFT output can be viewed as two real-valued matrices (the real-part matrix and the imaginary-part matrix) defined on the same (f,τ) grid. Furthermore, note that the analytic signal can be written as $x_{a}\left[n\right]=x\left[n\right]+i\hat{x}\left[n\right]$. Since STFT is a linear operator, we have

(17) $$\mathrm{STFT}\left\{x_{a}\left[n\right]\right\}=\mathrm{STFT}\left\{x\left[n\right]\right\}+i\;\mathrm{STFT}\left\{\hat{x}\left[n\right]\right\}.$$

Therefore, $Z_{r}$ and $Z_{i}$ correspond to the time–frequency projections of the original signal x[n] and its Hilbert-orthogonal component $\hat{x}\left[n\right]$, respectively. This indicates that the branch does not apply STFT only to a “real-part signal”; rather, a single transform of the complex analytic signal simultaneously produces both real and imaginary 2D time–frequency maps.

The complex-valued time–frequency map Z(t,f) is then used as a 2D complex input to a 2D complex-valued convolution module. This module contains two sets of learnable parameters for the real and imaginary parts of the complex kernels, corresponding to the “Real Filter” $V_{r}$ and “Imaginary Filter” $V_{i}$ in Figure 2. The complex-valued convolution kernel at layer *l* is

(18) $$V^{\left(l\right)}=V_{r}^{\left(l\right)}+iV_{i}^{\left(l\right)},\;l\in \left\{1,2,3\right\}.$$

Corresponding to the “Real Output” and “Imaginary Output” branches in the figure, given an input $U=U_{r}+iU_{i}$ and a kernel $V=V_{r}+iV_{i}$, the real and imaginary parts of the 2D complex convolution output Y=U∗V are

(19) $$Y_{r}=U_{r}*V_{r}-U_{i}*V_{i},\;Y_{i}=U_{r}*V_{i}+U_{i}*V_{r},$$

where ∗ denotes 2D convolution.

To enable the kernel to characterize structural variations in both local frequency neighborhoods and local time neighborhoods on the time–frequency plane, each 2D complex-valued kernel is parameterized as a Gaussian-windowed complex exponential and is defined along both the frequency and time directions:

(20) $$V_{k}^{\left(l\right)}\left(\Delta f,\Delta \tau \right)=\exp\;\left(-\frac{\Delta f^{2}}{2{\left(\sigma_{f,k}^{\left(l\right)}\right)}^{2}}-\frac{\Delta \tau^{2}}{2{\left(\sigma_{\tau ,k}^{\left(l\right)}\right)}^{2}}\right)\exp\;\left(i\;\left(2\pi \nu_{k}^{\left(l\right)}\Delta f+2\pi \mu_{k}^{\left(l\right)}\Delta \tau +\phi_{k}^{\left(l\right)}\right)\right).$$

Here, Δf and Δτ denote the local offsets along the frequency and time directions relative to the kernel center, defining the local receptive region on the time–frequency plane. The parameters $\sigma_{f,k}^{\left(l\right)}$ and $\sigma_{\tau ,k}^{\left(l\right)}$ control the effective ranges of the *k*-th kernel in layer *l* along the frequency and time directions, respectively. Their values determine the smoothness and effective support size in each direction and thus affect the time–frequency resolution characteristics. The first exponential term forms a 2D Gaussian window that locally constrains the kernel response, focusing it on a limited frequency neighborhood and time neighborhood. The parameters $\nu_{k}^{\left(l\right)}$ and $\mu_{k}^{\left(l\right)}$ are the oscillation-center parameters of the complex exponential along the frequency and time directions, respectively, describing the kernel’s selectivity to different frequency-change and time-change patterns. $\phi_{k}^{\left(l\right)}$ is a phase offset term that adjusts phase alignment in the kernel response. With this Gaussian-windowed complex-exponential parameterization, each 2D complex kernel can jointly model local amplitude-variation structures and phase-evolution characteristics on the time–frequency plane, enabling a unified characterization of complex time–frequency patterns.

The forward propagation of the three 2D complex convolution layers can be written recursively as

(21) $$Y^{\left(1\right)}=Z*V^{\left(1\right)},\;Y^{\left(2\right)}=Y^{\left(1\right)}*V^{\left(2\right)},\;Y^{\left(3\right)}=Y^{\left(2\right)}*V^{\left(3\right)}.$$

$Y^{\left(3\right)}$ is the complex-valued feature map output by the third layer. Its real part mainly reflects the response strength and energy variations of different frequency components within a local temporal range, whereas its imaginary part is more sensitive to phase variations and changes in relative timing relationships between frequency components.

As shown on the right side of the lower part of Figure 2, we perform amplitude–phase decomposition only on the third-layer complex output $Y^{\left(3\right)}$ to obtain an amplitude map and a phase map:

(22) $$A\left(t,f\right)=\left|Y^{\left(3\right)}\left(t,f\right)\right|,\;P\left(t,f\right)=∠Y^{\left(3\right)}\left(t,f\right).$$

The amplitude and phase features are concatenated along the channel dimension to form a joint time–frequency feature map:

(23) $$U_{tf}\left(t,f\right)=\left[A\left(t,f\right),\;P\left(t,f\right)\right].$$

Here, [·,·] denotes concatenation along the channel dimension, which jointly represents amplitude and phase information while keeping the time–frequency resolution unchanged.

To obtain a fixed-length time–frequency feature vector, as shown in Figure 2, we apply 2D global average pooling to $U_{tf}\left(t,f\right)$, i.e., we compute a global average over the entire T×F time–frequency plane for each channel:

(24) $$f_{tf}={\mathrm{GAP}}_{2D}\;\left(U_{tf}\left(t,f\right)\right),\;{\left(f_{tf}\right)}_{c}=\frac{1}{TF}\sum_{t=1}^{T}\sum_{f=1}^{F}U_{tf}\left(c,t,f\right).$$

Here, $U_{tf}\left(c,t,f\right)$ denotes the response of the joint time–frequency feature at channel *c* and the time–frequency point (t,f).

The time–frequency feature vector $f_{tf}$ is then passed through a linear transformation and normalized to obtain the time–frequency embedding vector of the modality:

(25) $$h_{tf}=W_{tf}f_{tf}+b_{tf},$$

where $W_{tf}$ and $b_{tf}$ are learnable parameters.

Finally, as shown by “Final Fusion/Concatenation” on the right side of Figure 2, the time-domain–phase branch output $h_{t}$ and the time–frequency branch output $h_{tf}$ are concatenated along the feature dimension:

(26) $$h=[h_{t},\;h_{tf}].$$

Here, [·,·] denotes vector concatenation. In this way, each modality obtains a unified representation that encodes both time-domain local response characteristics (amplitude–phase) and time–frequency structural variation information, providing a stable input for subsequent multimodal feature fusion and classification.

### 3.4. Feature Fusion: DLSAF

Multimodal features differ substantially in scale, spectral structure, and statistical characteristics. Simple feature concatenation limits the extraction of effective mutual information and reduces the overall signal-to-noise ratio. In addition, cross-modal response asynchrony and the dynamically non-stationary nature of signal quality further exacerbate the difficulty of feature alignment. Moreover, overly strong cross-modal consistency constraints may excessively compress complementary information across modalities and may also introduce misleading noise.

**Figure 3:**
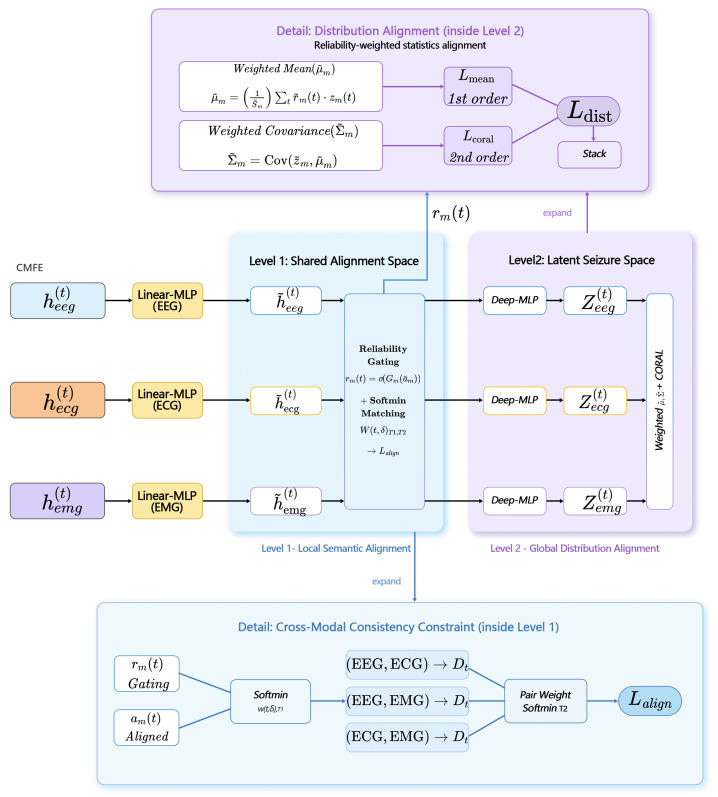
Architecture of the DLSAF module. The first level enforces cross-modal consistency in a shared alignment space through reliability gating and window-level softmin matching ($L_{cm}$). The second level aligns the mean and covariance of modality-specific features in a latent seizure space ($L_{dist}$).

To address these issues, this section proposes a Dual-Level Semantic Alignment Fusion module (DLSAF), as shown in Figure 3. DLSAF consists of two components: a cross-modal consistency constraint in an alignment space and distribution alignment in a shared latent space. Specifically, EEG, ECG, and EMG features are first projected into a unified alignment space through learnable Linear-MLP mappings. Within this space, a window-level softmin soft matching mechanism is introduced to accommodate cross-modal asynchrony. In addition, a modality-reliability gating mechanism adaptively weights the constraint strength across different time segments. This design suppresses the negative impact of low-quality modalities on the fused representation while improving robustness, yielding the cross-modal consistency loss $L_{cm}$. Next, the aligned modality representations are fed into a Deep-MLP to map them into a shared epilepsy-semantic latent space. Within this space, distribution-level alignment is performed based on reliability-weighted first-order statistics (mean) and second-order statistics (covariance), resulting in the distribution alignment loss $L_{dist}$. Finally, the latent representation sequences of the three modalities are stacked along the modality dimension to construct a three-dimensional feature block (3D block), which serves as the input to the subsequent 3D module for joint modeling along the time-modality-feature dimensions.

Let $h_{eeg}^{\left(t\right)}$, $h_{ecg}^{\left(t\right)}$, and $h_{emg}^{\left(t\right)}$ denote the EEG, ECG, and EMG modality feature vectors, respectively, obtained from the feature extraction module at the *t*-th time segment.

**(1)**  **Cross-Modal Consistency-Constrained Feature Fusion in the Alignment Space**

To align high-level semantic representations of different modalities along the temporal dimension, the features extracted from each modality at each time segment are first mapped into a shared alignment space of the same dimensionality. As illustrated by the Linear-MLP module in Figure 3, considering that the original feature dimensions and distributions may differ across modalities, a set of learnable linear projections is employed to map each modality into an alignment space of dimension $d_{h}$, formulated as:

$${\tilde{h}}_{\mathrm{eeg}}^{\left(t\right)}=W_{\mathrm{eeg}}h_{\mathrm{eeg}}^{\left(t\right)}+b_{\mathrm{eeg}},\;{\tilde{h}}_{\mathrm{ecg}}^{\left(t\right)}=W_{\mathrm{ecg}}h_{\mathrm{ecg}}^{\left(t\right)}+b_{\mathrm{ecg}},\;{\tilde{h}}_{\mathrm{emg}}^{\left(t\right)}=W_{\mathrm{emg}}h_{\mathrm{emg}}^{\left(t\right)}+b_{\mathrm{emg}}.$$

Here, $W_{\left(\cdot \right)}\in R^{d_{h}\times d}$ and $b_{\left(\cdot \right)}\in R^{d_{h}}$ are learnable parameters, and ${\tilde{h}}_{\left(\cdot \right)}^{\left(t\right)}\in R^{d_{h}}$ denotes the modality representation in the shared alignment space.

Considering that different modalities may exhibit temporal delays and asynchrony when reflecting the same epileptic state, and that signal quality dynamically fluctuates due to artifacts, missing data, and environmental noise, we adopt a window-level soft matching–reliability gating–modality-pair adaptive weighting strategy for consistency regularization within the alignment space. As shown in the Reliable Window Consistency Branch in Figure 3, we first define the temporal window set:

(27) $$K\left(t\right)=\left\{k|1\leq k\leq T,\;|k-t|\leq \Delta \right\},$$

where Δ denotes the window radius (measured in segments).

For any modality m∈{eeg,ecg,emg}, a reliability weight $r_{m}^{\left(t\right)}\in \left(0,1\right)$ is estimated at each time segment *t* through a learnable gating mechanism:

(28) $$r_{m}^{\left(t\right)}=\sigma \;\left(g_{m}\;\left({\tilde{h}}_{m}^{\left(t\right)}\right)\right),$$

where $g_{m}\left(\cdot \right)$ is implemented as a lightweight fully connected network and σ(·) denotes the Sigmoid function. This reliability weight is used to suppress the adverse influence of low-quality modalities or segments on the consistency constraint.

For each modality pair (m,n), using ${\tilde{h}}_{m}^{\left(t\right)}$ as the anchor, candidate segments from modality *n* within the window K(t) are softly matched. The reliability gating is directly incorporated into the matching distance, yielding the reliability-weighted window matching distance at segment *t*:

(29) $$D_{t}^{m,n}=\sum_{k\in K(t)}a_{t,k}^{m,n}\underset{\mathrm{reliability}\;\mathrm{gating}}{\underbrace{r_{m}^{\left(t\right)}r_{n}^{\left(k\right)}}}{∥{\tilde{h}}_{m}^{\left(t\right)}-{\tilde{h}}_{n}^{\left(k\right)}∥}_{2}^{2},\;\left(m,n\right)\in P,$$

where P={(eeg,ecg),(eeg,emg),(ecg,emg)} denotes the set of modality pairs. The coefficient $a_{t,k}^{m,n}$ is defined via a softmin operation within the window (controlled by temperature parameter τ):

(30) $$a_{t,k}^{m,n}=\frac{\exp\;\left(-{∥{\tilde{h}}_{m}^{\left(t\right)}-{\tilde{h}}_{n}^{\left(k\right)}∥}_{2}^{2}/\tau \right)}{\sum_{j\in K(t)}\exp\;\left(-{∥{\tilde{h}}_{m}^{\left(t\right)}-{\tilde{h}}_{n}^{\left(j\right)}∥}_{2}^{2}/\tau \right)}.$$

Here, τ>0 controls the sharpness of matching within the window. A smaller τ makes the softmin operation closer to hard minimum matching. This window-based soft matching allows cross-modal asynchrony: when the true semantic correspondence occurs at k≠t but satisfies |k−t|≤Δ, the constraint will focus on the most consistent candidate segment within the window, thereby suppressing gradient noise caused by forced mismatching.

Next, as illustrated by the $\mathrm{softmin}\to p_{t}^{m,n}$ branch in Figure 3, different modality pairs may exhibit varying consistency levels at the same segment *t*. Therefore, a softmin normalization is further applied across modality pairs over the set P to obtain pairwise weights:

(31) $$p_{t}^{m,n}=\frac{\exp(-\gamma D_{t}^{m,n})}{\sum_{(i,j)\in P}\exp\left(-\gamma D_{t}^{i,j}\right)},\;\left(m,n\right)\in P,$$

where γ>0 controls the sharpness of the weight distribution, assigning larger constraint weights to modality pairs with stronger consistency (smaller distance).

The cross-modal consistency loss at segment *t* is then defined as:

(32) $$L_{cm}^{\left(t\right)}=\sum_{(m,n)\in P}p_{t}^{m,n}\;D_{t}^{m,n},$$

and the overall consistency loss across all segments within a sample is computed as:

(33) $$L_{cm}=\frac{1}{T}\sum_{t=1}^{T}L_{cm}^{\left(t\right)}.$$

By minimizing $L_{cm}$, the model encourages different modalities to form a stable and coherent structure around the same epileptic semantic representation while allowing cross-modal asynchrony. Meanwhile, the reliability gating mechanism mitigates the negative impact of low-quality modalities on the fused representation, thereby improving multimodal synergy and robustness.

After cross-modal consistency regularization, the temporally aligned feature sequences of each modality are expressed as:

$${\tilde{H}}_{\mathrm{eeg}}={\left\{{\tilde{h}}_{\mathrm{eeg}}^{\left(t\right)}\right\}}_{t=1}^{T},\;{\tilde{H}}_{\mathrm{ecg}}={\left\{{\tilde{h}}_{\mathrm{ecg}}^{\left(t\right)}\right\}}_{t=1}^{T},\;{\tilde{H}}_{\mathrm{emg}}={\left\{{\tilde{h}}_{\mathrm{emg}}^{\left(t\right)}\right\}}_{t=1}^{T}.$$

**(2)** 
**Shared Latent-Space Alignment for Feature Fusion (Shared Latent Seizure Space)**

In the alignment space feature fusion step above, the three modality features are mapped into the same alignment space, and a cross-modal consistency loss $L_{cm}$ is constructed via window-level soft matching, reliability gating, and modality-pair adaptive weighting. This achieves segment-level local semantic alignment along the temporal dimension. However, because different modalities may still exhibit global shifts in statistical distributions and correlation structures, relying only on local consistency constraints can lead to different distributional patterns in the latent representation space. This may in turn degrade generalization across subjects and under varying noise conditions. To further improve modality invariance at the distribution level, as shown in the pipeline “Deep-MLP → weighted mean/covariance → dist → $L_{dist}$” on the right side of Figure 3, we introduce a shared latent seizure space. In this space, we align the first-order statistics (mean) and second-order statistics (covariance) of modality-specific latent representations to obtain a more compact epilepsy-semantic representation with consistent distributions.

For each time segment t∈{1,…,T}, the modality feature ${\tilde{h}}_{m}^{\left(t\right)}\in R^{d_{h}}$ in the alignment space is fed into the modality-specific Deep-MLP $f_{m}\left(\cdot \right)$ in Figure 3, yielding the shared latent-space representation $z_{m}^{\left(t\right)}\in R^{d_{z}}$:

(34) $$z_{m}^{\left(t\right)}=f_{m}\;\left({\tilde{h}}_{m}^{\left(t\right)}\right),\;m\in \left\{\mathrm{eeg},\mathrm{ecg},\mathrm{emg}\right\}.$$

To ensure that global distribution estimation is dominated by high-quality segments, we reuse the reliability weights $r_{m}^{\left(t\right)}\in \left(0,1\right)$ obtained in the alignment space feature fusion step above, and define the normalized weight sum as

(35) $$S_{m}=\sum_{t=1}^{T}r_{m}^{\left(t\right)}+\varepsilon ,$$

where ε>0 is a numerical stability term. Then, as shown in the “weighted mean/weighted covariance“ modules in Figure 3, we compute the reliability-weighted mean and covariance of modality *m* in the shared latent space. The weighted mean vector is defined as

(36) $${\bar{z}}_{m}=\frac{1}{S_{m}}\sum_{t=1}^{T}r_{m}^{\left(t\right)}\;z_{m}^{\left(t\right)},\;m\in \left\{\mathrm{eeg},\mathrm{ecg},\mathrm{emg}\right\}.$$

To further align distributional shapes, we introduce the reliability-weighted covariance matrix:

(37) $$C_{m}=\frac{1}{S_{m}}\sum_{t=1}^{T}r_{m}^{\left(t\right)}\left(z_{m}^{\left(t\right)}-{\bar{z}}_{m}\right){\left(z_{m}^{\left(t\right)}-{\bar{z}}_{m}\right)}^{⊤},\;m\in \left\{\mathrm{eeg},\mathrm{ecg},\mathrm{emg}\right\}.$$

Based on these statistics, as indicated by “dist →$L_{dist}$” in Figure 3, we jointly characterize modality distribution shifts using differences in means and covariances. The first-order alignment loss is

(38) $$L_{\mu }=∥{\bar{z}}_{\mathrm{eeg}}-{\bar{z}}_{\mathrm{ecg}}{∥}_{2}^{2}+∥{\bar{z}}_{\mathrm{eeg}}-{\bar{z}}_{\mathrm{emg}}{∥}_{2}^{2}+{∥{\bar{z}}_{\mathrm{ecg}}-{\bar{z}}_{\mathrm{emg}}∥}_{2}^{2}.$$

The second-order (CORAL) alignment loss is

(39) $$L_{cor}=∥C_{\mathrm{eeg}}-C_{\mathrm{ecg}}{∥}_{F}^{2}+∥C_{\mathrm{eeg}}-C_{\mathrm{emg}}{∥}_{F}^{2}+{∥C_{\mathrm{ecg}}-C_{\mathrm{emg}}∥}_{F}^{2},$$

where ${∥\cdot ∥}_{F}$ denotes the Frobenius norm. Finally, the distribution-level alignment loss is defined as

(40) $$L_{dist}=L_{\mu }+\beta L_{cor},$$

where β≥0 balances the relative strength between mean alignment and covariance alignment. With this distribution alignment mechanism, while ensuring that reliable segments dominate the estimation, the model can simultaneously reduce global center shifts and distribution-shape differences across modalities, thereby producing a more compact and modality-invariant epilepsy-semantic representation.

After shared latent-space alignment, as shown in the stacking module in Figure 3, the latent representation sequence of modality *m* is denoted as $Z_{m}={\left\{z_{m}^{\left(t\right)}\right\}}_{t=1}^{T}$. To facilitate subsequent joint modeling across time–feature–modality by the 3D module, the latent representations of the three modalities are stacked along the modality dimension to form a 3D feature block:

(41) $$F=\mathrm{Stack}\left(Z_{\mathrm{eeg}},Z_{\mathrm{ecg}},Z_{\mathrm{emg}}\right)\in R^{T\times d_{z}\times n},\;n=3.$$

This feature block serves as the input to the subsequent 3D convolution/attention classification module.

Finally, to enable end-to-end optimization for the overall task, we denote the prediction loss as $L_{cls}$. The total training loss is:

(42) $$L=L_{cls}+\lambda_{1}L_{cm}+\lambda_{2}L_{dist}=L_{cls}+\lambda_{1}L_{cm}+\lambda_{2}\left(L_{\mu }+\beta L_{cor}\right),$$

where $L_{cm}$ is the cross-modal consistency loss defined in the alignment space feature fusion step above, and $\lambda_{1}\geq 0$ and $\lambda_{2}\geq 0$ balance the contributions of local consistency regularization and distribution-level alignment regularization in the overall objective.

### 3.5. Classifier (3DCSA)

After applying the cross-modal consistency constraint and the shared latent-space alignment, the latent representations of EEG, ECG, and EMG from different time segments are stacked to form a 3D fused feature block:

(43) $$F\in R^{T\times d_{z}\times n},$$

where *T* denotes the number of time segments, $d_{z}$ is the feature dimensionality in the shared latent space, and *n* is the number of modalities (in this study, n=3 for EEG, ECG, and EMG). This 3D block encodes multimodal epilepsy-semantic information along the time, feature, and modality dimensions and serves as the input to the subsequent classifier.

**Figure 4:**
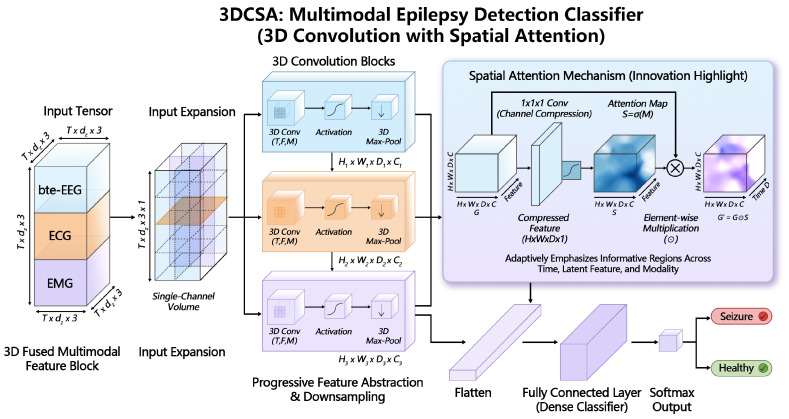
Architecture of the 3DCSA classifier. Stacked 3D convolutional blocks jointly model the temporal, feature, and modality dimensions of the fused feature block. A spatial attention map then reweights informative regions before a fully connected Softmax layer outputs the seizure probability.

To fully exploit the joint structure of F across these three dimensions, we design a multimodal feature classifier based on a 3D convolutional neural network with a spatial attention mechanism, termed 3D Convolution with Spatial Attention (3DCSA), as illustrated in Figure 4. Specifically, F is treated as a 3D feature volume of size $T\times d_{z}\times n$, and it is further expanded to a single-channel tensor along the channel dimension:

(44) $$X\in R^{T\times d_{z}\times n\times 1},$$

which is used as the input to the 3D convolutional network.

3DCSA is composed of multiple stacked 3D convolutional blocks. Each block includes a 3D convolution layer, a nonlinear activation function, and a 3D pooling layer. The 3D convolution kernels slide simultaneously along the time, feature, and modality dimensions, enabling the network to capture (i) dynamic changes across time segments, (ii) coupling relationships among latent features, and (iii) correlation patterns across modalities. The subsequent 3D pooling operations progressively reduce the volume size while preserving higher-level semantic representations. After multiple layers of 3D convolution and pooling, a high-dimensional feature map is obtained:

(45) $$G\in R^{H\times W\times D\times C},$$

where *H*, *W*, and *D* correspond to the downsampled time, feature, and modality/spatial dimensions, respectively, and *C* denotes the number of channels.

To further enhance the model’s ability to focus on key regions, 3DCSA introduces a spatial attention mechanism on the high-level feature map. A 1×1×1 3D convolution is used to compress channels in G, producing a single-channel attention feature map M. A Sigmoid activation is then applied to generate the spatial attention weights:

(46) S=σ(M),

where each element in S lies in [0,1] and indicates the importance of the corresponding spatial location (i.e., a specific combination of time segment, latent feature channel, and modality). The attention map is multiplied element-wise with the original feature map:

(47) $$G^{\prime }=G⊙S,$$

thereby amplifying regions that are more relevant to epilepsy-state discrimination while suppressing irrelevant or noisy regions.

Finally, the weighted feature map $G^{\prime }$ is flattened into a vector and fed into a fully connected layer and a Softmax classifier to output the class probabilities for different epilepsy states. By combining the complex-domain features extracted by CMFE, the fused representations obtained via cross-modal consistency and shared latent-space alignment, and the joint time–feature–modality modeling capability of 3DCSA, the model achieves higher detection accuracy and improved robustness on the SeizeIT2 [37] dataset.

## 4. Experimental and Results

### 4.1. Dataset

All experiments in this study were conducted on the public SeizeIT2 [37] benchmark dataset. SeizeIT2 is a multicenter wearable multimodal epilepsy dataset. Its public subset contains 125 patients with focal epilepsy from five epilepsy monitoring centers in Europe. The dataset contains approximately 11,640 h of wearable monitoring data and 886 focal seizure events. The recordings were collected in both home and hospital monitoring environments and provide multiple modalities, including behind-the-ear EEG, ECG, EMG, and motion signals. Therefore, SeizeIT2 is suitable for automatic seizure-event detection under long-term monitoring conditions.

To avoid data leakage, the dataset was divided at the subject level before sliding-window segmentation and temporal sample construction. We adopted the subject-independent holdout split recommended by SeizeIT2. The training set contained subjects from sub-001 to sub-096. The test set contained subjects from sub-098 to sub-125. Subject sub-097 was excluded because its multimodal recordings were incomplete and could not be used to construct the EEG, ECG, and EMG inputs required by this study. All recordings, window segments, and temporal samples from the same subject were assigned to the same data subset. Therefore, no subject appeared in both the training and test sets.

The experiments used three signal modalities: dual-channel EEG, single-channel ECG, and single-channel EMG. This study focuses on seizure-event detection rather than patient-level epilepsy diagnosis. Therefore, the model did not determine whether a subject had epilepsy by majority voting over all segments from that subject. Instead, it first generated window-level seizure probabilities. These probabilities were then converted into predicted seizure events through a unified post-processing procedure and matched with expert-annotated seizure events along the time axis.

### 4.2. Evaluation Metrics

The evaluation uses five metrics to assess model performance: sensitivity (Sens), false alarms per hour (FA/h), HMS, area under the Sens–FA/h curve (AUSF), and area under the receiver operating characteristic curve (AUROC). Sens, FA/h, and AUSF measure seizure-event-level performance, whereas AUROC measures window-level performance. Sens reflects the seizure detection ability of the model. FA/h reflects the false alarm level during non-seizure periods. HMS provides a single-threshold composite score that summarizes the trade-off between sensitivity and false alarms. AUSF evaluates the overall trade-off between sensitivity and false alarms under different thresholds. AUROC evaluates the overall discriminative ability of the model between seizure and non-seizure samples.

In the experiments, the evaluation segments raw signals using a sliding window with a length of 2 s and a step size of 1 s. The signal sampling rate is 256 Hz. Therefore, each window contains 512 samples. For the *i*-th window, the model outputs a seizure probability $p_{i}\in \left[0,1\right]$, forming a window-level probability sequence ${\left\{p_{i}\right\}}_{i=1}^{N}$. The evaluation computes AUROC directly from the window-level prediction probabilities and their corresponding labels. This computation does not involve threshold selection or post-processing.

For Sens, FA/h, and AUSF, the post-processing procedure first converts the window-level probability sequence into a list of predicted seizure events. The event-level matching procedure then matches the predicted seizure events with expert-annotated seizure events along the time axis. This study adopts the any-overlap criterion for event-level matching. Let the predicted event be ${\hat{e}}_{j}=\left[{\hat{t}}_{j}^{s},{\hat{t}}_{j}^{e}\right]$, and let the true seizure event be $e_{k}=\left[t_{k}^{s},t_{k}^{e}\right]$. Here, *t* denotes the temporal position of an event on the recording timeline. Superscripts *s* and *e* denote the start time and end time of an event, respectively. Subscripts *j* and *k* denote the indices of the predicted event and the true event, respectively. ${\hat{e}}_{j}$ denotes the model prediction, and $e_{k}$ denotes the expert annotation.

If the two events satisfy

$$\min\left({\hat{t}}_{j}^{e},t_{k}^{e}\right)-\max\left({\hat{t}}_{j}^{s},t_{k}^{s}\right)>0,$$

the evaluation regards the predicted event as matching the true event and counts it as a true positive (TP). If no predicted event covers a true seizure event, the evaluation counts the true seizure event as a false negative (FN). Sensitivity is defined as

$$\mathrm{Sens}=\frac{\mathrm{TP}}{\mathrm{TP}+\mathrm{FN}}.$$

If a predicted event does not overlap with any true seizure event, the evaluation counts it as a false positive (FP). Let the total recording duration be $T_{\mathrm{rec}}$, measured in seconds. FA/h is defined as

$$FA/h=\frac{\mathrm{FP}\times 3600}{T_{\mathrm{rec}}}.$$

Sens and FA/h characterize the seizure detection ability and false alarm control ability of the model, respectively. Together, they form key indicators for evaluating the clinical usability of the model. To provide an additional single-threshold summary of the trade-off between sensitivity and false alarm burden, this study also reports HMS as a supplementary composite metric. Following the definition introduced in the SeizeIT2 seizure detection challenge, HMS is defined as

HMS=Sens(%)−0.4×FA/h.

A higher HMS indicates a better balance between seizure detection sensitivity and false alarm control at the fixed decision threshold.

AUSF is calculated from the Sens–FA/h curve. First, the detection threshold is uniformly divided into 51 values within the interval [0,1], denoted as ${\left\{\theta_{m}\right\}}_{m=0}^{50}$. The model first outputs the seizure probability sequence for each test recording. Then, each threshold $\theta_{m}$ serves as the decision threshold to convert the probability sequence into a binary prediction sequence, followed by unified post-processing. The evaluation then obtains the Sens–FA/h curve for each test recording. Next, the curves of all recordings are interpolated onto a unified horizontal axis x∈[0,200]. The evaluation averages the interpolated curves over all recordings containing seizures and obtains the mean sensitivity function $\bar{s}\left(x\right)$. AUSF is defined as

$$\mathrm{AUSF}=\frac{1}{200}\int_{0}^{200}\bar{s}\left(x\right)\;dx.$$

Its value ranges from 0 to 1. A higher AUSF indicates that the model achieves a better balance between sensitivity and false alarm control under different thresholds. The main results are reported at the fixed threshold θ=0.5, whereas AUSF evaluates the overall performance of the model under different thresholds.

The post-processing procedure contains three steps. The boundary parameter is set to Δ=10s. First, the procedure binarizes the window-level probability sequence according to the threshold:

$$b_{i}=1\left[p_{i}>\theta \right].$$

Then, the procedure fills and merges segments whose gaps between adjacent predicted events are shorter than 0.2Δ=2s. This step reduces event fragmentation caused by short interruptions. Finally, the procedure removes isolated predicted events whose durations are shorter than 0.8Δ=8s. This step suppresses false detections caused by transient noise.

Before post-processing, the evaluation controls the signal quality of each EEG window. First, it calculates the root mean square (RMS) amplitude of each channel. If the RMS of any channel falls below 13μV or exceeds 150μV [37], the procedure sets the prediction probability of the corresponding window to zero. This strategy reduces false detections caused by low-quality signals and improves the reliability of event-level detection. Because the seizure-window prevalence in the test set is extremely low (about 0.096%, i.e., roughly 1:1041), the window-level AUROC is only a weak proxy for event-level clinical utility. We therefore regard AUROC as an auxiliary window-level diagnostic, and use the event-level metrics (Sens, FA/h, AUSF, and HMS) as the primary criteria for clinical usability.

### 4.3. Training Configuration

This section sets the following training strategies and hyperparameters to ensure stable training and reproducible results. The model uses the Adam optimizer with an initial learning rate of $\eta_{0}=1\times 10^{-3}$ and a weight decay of $\lambda_{w}=1\times 10^{-4}$. The learning rate follows the StepLR strategy. Specifically, the learning rate is multiplied by a decay factor of 0.5 every 10 epochs, with a lower bound of $\eta_{\min}=1\times 10^{-5}$. The batch size is set to 32, and the maximum number of training epochs is set to 100. The gradient clipping threshold is set to 1.0. The random seed is fixed at 42.

The total loss consists of three components:

$$L=L_{\mathrm{cls}}+\lambda_{1}L_{cm}+\lambda_{2}L_{dist}.$$

Here, $L_{\mathrm{cls}}$ denotes the Focal Loss with γ=2.0. The dynamic positive-sample weight is defined as

$$w_{\mathrm{pos}}=\frac{n_{\mathrm{neg}}}{n_{\mathrm{pos}}}.$$

$L_{cm}$ denotes the cross-modal consistency loss, and $L_{dist}$ denotes the distribution alignment loss. The loss weights are set to $\lambda_{1}=\lambda_{2}=50$. The hidden dimension is set to $d_{h}=128$, and the alignment-space dimension is set to $d_{z}=64$. The dropout rates of the convolutional layer, alignment layer, and classification layer are set to 0.1, 0.3, and 0.5, respectively.

### 4.4. Comparison with Baseline Methods

**Figure 5:**
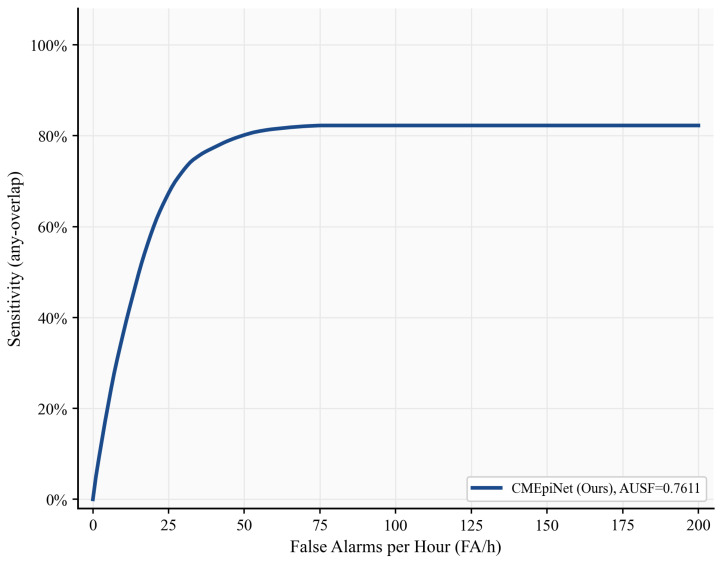
Threshold-dependent sensitivity–FA/h curve of CMEpiNet on the SeizeIT2 test set.

**Figure 6:**
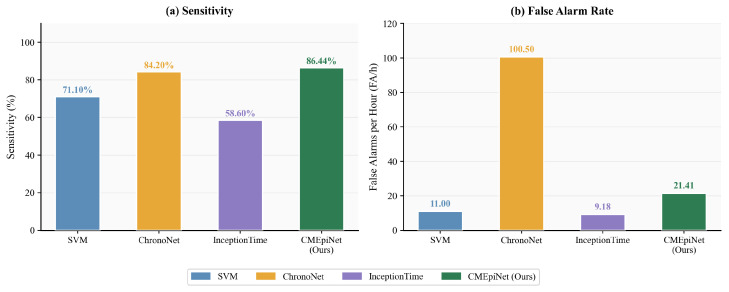
Comparison of sensitivity and FA/h on the SeizeIT2 test set.

To evaluate the performance of the proposed method, CMEpiNet was compared with the official SVM and ChronoNet benchmark results reported in the SeizeIT2 dataset paper. The SVM baseline represents a traditional feature-engineering-based machine-learning method. This baseline was adapted from the behind-the-ear EEG seizure detection framework proposed by Vandecasteele et al. [38]. In the official SeizeIT2 benchmark, the EEG signals were first filtered and then segmented into 2 s windows with 50% overlap. Each window segment was represented by 42 handcrafted features and classified using a support vector machine with a radial basis function kernel. ChronoNet represents the deep-learning baseline. It is a convolutional-recurrent neural network originally proposed by Roy et al. [39] for abnormal EEG identification. ChronoNet takes EEG temporal segments as input and combines one-dimensional convolutional layers with gated recurrent units to model temporal patterns in EEG signals. The SVM and ChronoNet results used in this study were taken from the official SeizeIT2 benchmark, rather than being independently reimplemented or reoptimized in this work. In addition, InceptionTime [40] was included as a typical deep multivariate time-series classification baseline and was evaluated under the same SeizeIT2-based protocol. InceptionTime is a convolutional time-series classification network composed of stacked Inception modules with multi-scale temporal convolution kernels and residual connections. In this study, we used a single InceptionTime network rather than the ensemble version. The EEG, ECG, and EMG inputs were concatenated along the channel dimension to form a four-channel multivariate time series for each 2 s window. To ensure a fair comparison with CMEpiNet, InceptionTime was evaluated using the same subject-independent SeizeIT2 split, input modalities, preprocessing pipeline, and event-level evaluation protocol. The training protocol was also aligned with that of CMEpiNet, including Focal Loss, Adam optimization, batch size, learning-rate schedule, gradient clipping, early stopping, random seed, and validation-based model selection. Table 1 reports the quantitative results of the compared methods on the SeizeIT2 test set. Figure 5 shows the threshold-dependent Sens–FA/h curve of CMEpiNet on the SeizeIT2 test set, which is used to analyze the operating characteristics of the proposed model under different decision thresholds. The quantitative and visual comparisons among the compared methods are provided in Table 1 and Figure 6. Figure 6 compares the Sens and FA/h values of the compared methods on the SeizeIT2 test set.

Compared with SVM, CMEpiNet improves sensitivity from 71.10% to 86.44%, with an increase of 15.34%. It also improves AUSF from 0.7229 to 0.7611, with an increase of 3.82%. SVM achieves a low FA/h of 11.00 and therefore shows a certain advantage in false alarm control. However, its sensitivity is low, indicating a more serious missed-detection problem. In contrast, CMEpiNet significantly improves seizure detection ability while maintaining an acceptable false alarm level. The improvement in AUSF further indicates that this advantage is not limited to a single threshold but remains across a broader range of operating points. HMS also increased from 66.70 for SVM to 77.88 for CMEpiNet, further indicating that CMEpiNet achieves a better overall trade-off between sensitivity and false-alarm burden at the fixed decision threshold.

ChronoNet achieves a sensitivity of 84.20% and an FA/h of 100.50, indicating an extremely high false alarm level. This result means that the model triggers more than 100 false alarms per hour on average, which cannot meet the requirements of practical clinical or home-monitoring scenarios. In contrast, CMEpiNet improves sensitivity from 84.20% to 86.44%, with an increase of 2.24%. It also reduces FA/h from 100.50 to 21.41. These results show that CMEpiNet improves seizure detection ability while substantially improving false alarm control. HMS also increased from 44.00 for ChronoNet to 77.88 for CMEpiNet, indicating that CMEpiNet achieves a more favorable balance between seizure detection performance and false-alarm control at the fixed operating point.

Compared with InceptionTime, CMEpiNet improves sensitivity from 58.60% to 86.44%, corresponding to an increase of 27.84 percentage points. InceptionTime achieves a low FA/h of 9.18, indicating better false-alarm control, but its substantially lower sensitivity suggests a higher risk of missed seizures in this task. Although CMEpiNet has a higher FA/h than InceptionTime, its HMS increases from 54.93 to 77.88. This result indicates that CMEpiNet achieves substantially higher seizure detection sensitivity with a moderate increase in false alarms, leading to a better overall trade-off at the fixed decision threshold.

### 4.5. Sensitivity Analysis by Seizure Type

To examine whether the overall sensitivity masked subtype-specific detection deficits, we performed a seizure-type stratified analysis (Table 2). CMEpiNet detected all focal to bilateral tonic–clonic (FBTC) seizures (55/55, 100.00%), and achieved sensitivities of 84.17% (101/120) for focal impaired awareness (FIA) seizures and 78.95% (45/57) for focal aware (FA) seizures. Four additional focal seizures were not further subclassified, of which three were detected. Because this category contained only four events, we report it for completeness without further statistical inference.

The subtype-specific results suggest a clinically plausible gradient in detection difficulty. Sensitivity was highest for FBTC seizures and decreased for FIA and FA seizures, consistent with the weaker motor and autonomic manifestations of less severe focal seizures. This pattern is also consistent with wearable multimodal detection, where FA seizures may provide weaker ECG and EMG evidence than FBTC seizures. Nevertheless, the per-type results do not suggest a complete failure in any major subtype: all clinically high-risk FBTC seizures were detected, and the more subtle FA seizures still reached a sensitivity of 78.95%.

This analysis also helps interpret the observed window-level AUROC. The missed detections were mainly from FA and FIA seizures, which may show weaker cross-modal signatures within individual 2 s windows. Their lower window-level separability may contribute to a weaker overall probability ranking and thus to the non-superior AUROC of CMEpiNet. In contrast, by leveraging multimodal fusion and event-level post-processing, CMEpiNet still detected a substantial proportion of seizure events. These results support our interpretation that the main advantage of CMEpiNet lies in event-level seizure detection rather than window-level probability ranking.

### 4.6. Ablation Study

**Figure 7:**
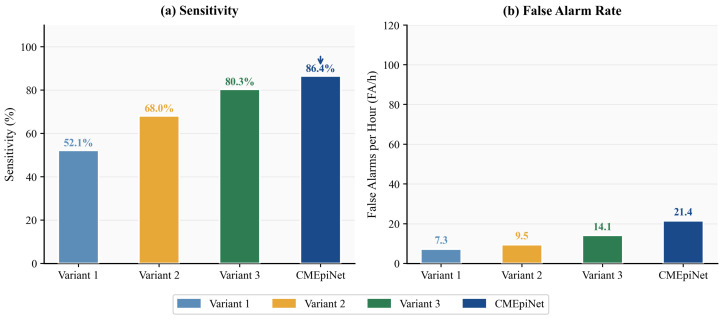
Ablation study of CMEpiNet on the SeizeIT2 test set in terms of sensitivity and FA/h.

The ablation experiments on the SeizeIT2 dataset verify the effectiveness of the CMFE and DLSAF modules. For this purpose, the experiments construct three ablation variants. Table 3 reports the results of all variants on the SeizeIT2 test set. Figure 7 compares Sensitivity and FA/h.

Variant 1, RealConv+Linear, uses a three-layer real-valued Conv1d branch to extract features and uses linear projection for modality mapping. It does not introduce any cross-modal alignment loss.

Variant 2, CMFE+Linear, uses the dual-branch complex-valued CMFE to extract multimodal features and uses linear projection for modality mapping. It does not introduce the cross-modal soft-window consistency constraint.

Variant 3, CMFE+Linear+$L_{cm}$, uses the dual-branch complex-valued CMFE to extract multimodal features and introduces the cross-modal soft-window consistency constraint.

CMEpiNet further introduces shared latent-space alignment based on Variant 3 and forms the complete dual-level semantic alignment fusion mechanism.

After introducing the dual-branch complex-valued CMFE, sensitivity increases from 52.15% to 68.04%. AUSF increases from 0.7597 to 0.7786. These results show that complex-valued feature extraction significantly enhances the seizure detection ability of the model. The time-domain phase branch constructs analytic signals through the Hilbert transform and jointly models instantaneous amplitude and phase information. The time–frequency branch computes the STFT using the original signal and its Hilbert transform, thereby preserving a more complete spectral phase structure in the time-frequency plane. These designs enhance the model’s ability to represent seizure-related time–frequency patterns.

After introducing the cross-modal consistency constraint based on Variant 2, sensitivity increases from 68.04% to 80.26%. This result shows that the cross-modal consistency constraint further improves the seizure detection rate. This module first uses reliability gating to estimate the credibility of each modality at local time steps. It then uses the soft-window matching loss to learn temporally consistent cross-modal representations. This mechanism allows the model to use information from other modalities for compensation when a single modality is disturbed or ambiguous, thereby reducing missed detections. Meanwhile, AUSF decreases from 0.7786 to 0.7620, and FA/h increases from 9.46 to 14.13. Although Level-1 alignment improves the model’s sensitivity to subtle seizure patterns, it also increases false activation in some non-seizure segments. This result reflects the inherent trade-off between sensitivity and false alarms.

After further introducing shared latent-space alignment based on Variant 3, sensitivity increases from 80.26% to 86.44%, with an increase of 6.18%. This result shows that global distribution alignment further enhances the seizure detection ability of the model. This module imposes alignment constraints on the mean vectors and covariance matrices of the three modalities in the feature space, namely $L_{dist}$. This constraint encourages the semantic representations of EEG, ECG, and EMG to become more consistent in the overall distribution, thereby reducing representation shifts caused by modality heterogeneity. Although the difference in AUSF between Variant 3 and the full model is small, with values of 0.7620 and 0.7611, sensitivity continues to improve. This indicates that shared latent-space alignment makes a substantive contribution to seizure detection ability.

Based on the ablation results in Table 3, the following conclusions can be drawn. As the core modules are gradually introduced, the model sensitivity continuously improves, reaching 52.15%, 68.04%, 80.26%, and 86.44%, respectively. From the basic baseline to the full model, sensitivity increases by 34.29%, indicating that each core module effectively improves seizure detection ability. Meanwhile, FA/h also increases from 7.26 to 9.46, 14.13, and 21.41. This indicates that the model produces more false alarms while improving sensitivity. As the feature representation ability becomes stronger, the model becomes more sensitive to weak seizure patterns and also produces a higher false alarm level. This result reflects the trade-off between improved detection ability and increased false alarms, and it should not be simply interpreted as performance degradation. On the other hand, the AUSF values of all ablation variants are close to or higher than 0.76. This indicates that the performance changes do not only reflect the movement of a single-threshold operating point but also maintain relatively stable comprehensive performance across thresholds.

### 4.7. Robustness Experiments

To evaluate the robustness of CMEpiNet under input perturbations, we conducted robustness experiments. The baseline model corresponded to Variant 1 in the preceding ablation study, namely RealConv+Linear. This model uses a three-layer real-valued 1D convolutional branch for multimodal feature extraction and linear projection for modality mapping. RealConv+Linear does not include the complex-valued multimodal feature extraction module (CMFE), the Dual-Level Semantic Alignment Fusion module (DLSAF), or the 3D Convolution with Spatial Attention (3DCSA) classifier incorporated in CMEpiNet. Specifically, it lacks the cross-modal consistency constraint and the distribution-level alignment in the shared latent space introduced by DLSAF. Therefore, this comparison was used to analyze the robustness gains contributed by these core modules under input perturbations. AUROC and AUSF were used to evaluate model robustness under perturbation conditions.

The performance change caused by perturbation is defined as the difference between the metric value under the baseline condition and that under the perturbation condition for the same model:

$$\Delta M=M_{\mathrm{baseline}}-M_{\mathrm{perturbed}},$$

where *M* denotes AUROC or AUSF. If ΔM>0, the performance decreases after perturbation. If ΔM=0, the metric remains unchanged before and after perturbation. If ΔM<0, the metric after perturbation is slightly higher than the baseline value.

Noise perturbation is applied after window segmentation. The experiments randomly select 20% and 30% of the windows and replace the selected EEG, ECG, and EMG windows with Gaussian noise of equal power. The noise intensity of each channel is determined by the standard deviation of the original window in that channel. This setting simulates different degrees of signal quality degradation.

Temporal-shift perturbation is applied before window segmentation. To simulate acquisition delay or temporal asynchrony between auxiliary modalities and EEG, the experiments use EEG as the temporal reference and apply temporal shifts only to ECG and EMG. The shift magnitudes are set to 128, 256, and 512 samples. Since the sampling rate is 256 Hz, these three shift magnitudes correspond to temporal offsets of 0.5 s, 1.0 s, and 2.0 s, respectively.

Table 4 reports the performance degradation under each perturbation condition.

CMEpiNet shows small AUROC degradation under all five perturbation conditions, ranging from 0.0011 to 0.0019. Its AUSF remains unchanged from the baseline value. The baseline model shows a more obvious AUROC decrease under noise perturbation. Its AUSF also shows different degrees of degradation under both noise perturbation and temporal-shift perturbation.

#### 4.7.1. Analysis of Noise Perturbation Results

Under noise perturbation, the AUROC of CMEpiNet decreases from the baseline value of 0.6631 to 0.6612 and 0.6613, corresponding to degradation values of 0.0019 and 0.0018, respectively. The AUSF of CMEpiNet remains 0.7611 under both 20% and 30% window noise replacement, corresponding to a degradation value of 0.0000 in both cases.

The AUROC of the baseline model decreases from the baseline value of 0.6962 to 0.6894 and 0.6871, corresponding to degradation values of 0.0068 and 0.0091, respectively. Its AUSF decreases from the baseline value of 0.7597 to 0.7462 and 0.7283, corresponding to degradation values of 0.0135 and 0.0314, respectively.

Under random window noise replacement, CMEpiNet shows a smaller performance decrease relative to its own baseline. Under the 30% window noise replacement condition, the AUSF degradation of the baseline model reaches 0.0314. This result shows that CMEpiNet has more stable event-level detection performance under signal quality degradation.

#### 4.7.2. Analysis of Temporal-Shift Perturbation Results

Under temporal-shift perturbation, the AUROC of CMEpiNet changes from the baseline value of 0.6631 to 0.6619, 0.6620, and 0.6618, corresponding to degradation values of 0.0012, 0.0011, and 0.0013, respectively. The AUSF of CMEpiNet remains 0.7611 under the 0.5 s, 1.0 s, and 2.0 s auxiliary-modality temporal-shift conditions.

The AUROC of the baseline model changes from the baseline value of 0.6962 to 0.6966, 0.6958, and 0.6954, corresponding to degradation values of −0.0004, 0.0004, and 0.0008, respectively. Under the 0.5 s auxiliary-modality temporal-shift condition, the AUROC of the baseline model is slightly higher than the baseline value, and therefore the degradation value is negative. The AUSF of the baseline model decreases from the baseline value of 0.7597 to 0.7536, 0.7506, and 0.7469, corresponding to degradation values of 0.0061, 0.0091, and 0.0128, respectively.

Both models maintain relatively stable AUROC under auxiliary-modality temporal shifts. However, the AUSF of the baseline model decreases as the temporal-shift magnitude increases. The AUSF of CMEpiNet shows no visible degradation under the three temporal-shift conditions. This indicates that its event-level detection performance has stronger stability under temporal asynchrony of auxiliary modalities.

#### 4.7.3. Robustness Analysis

**Figure 8:**
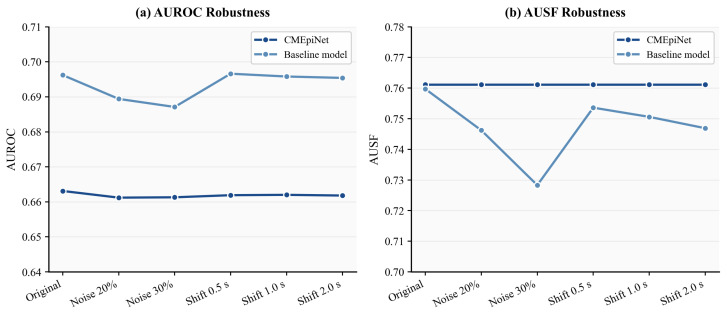
AUROC and AUSF trends of CMEpiNet and the baseline model under different perturbation conditions.

Figure 8 shows the trends of AUROC and AUSF for the two models under different perturbed test sets. Figure 8a shows that the AUROC curve of CMEpiNet remains nearly horizontal under all perturbation conditions, with values consistently maintained between 0.6612 and 0.6631. This indicates that perturbations have little influence on its window-level discriminative ability. The baseline model shows a more obvious decrease under noise perturbation. Figure 8b shows that the AUSF of CMEpiNet remains 0.7611 under all conditions, and its curve stays stable. In contrast, the AUSF of the baseline model decreases under both noise perturbation and temporal-shift perturbation. This figure indicates that CMEpiNet achieves more stable performance under different perturbation conditions, especially in terms of AUSF.

**Figure 9:**
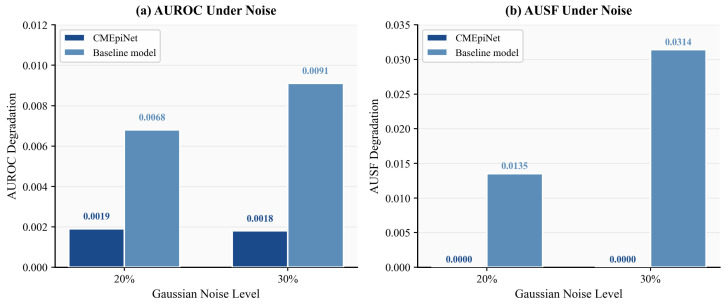
Performance degradation under window-level Gaussian noise replacement.

Figure 9 shows the performance degradation of the two models relative to their own baselines under window-level Gaussian noise replacement. The degradation value is defined as the baseline performance minus the perturbed performance. Therefore, a larger value indicates that the model is more strongly affected by perturbation. Figure 9a shows that under 20% and 30% window noise replacement, the AUROC degradation values of CMEpiNet are 0.0019 and 0.0018, whereas those of the baseline model are 0.0068 and 0.0091. Figure 9b shows that the AUSF degradation values of CMEpiNet are 0.0000 under both noise conditions. In contrast, the degradation values of the baseline model are 0.0135 and 0.0314. These results show that CMEpiNet exhibits a smaller relative performance decrease under signal quality degradation and achieves more stable robustness in terms of AUSF.

**Figure 10:**
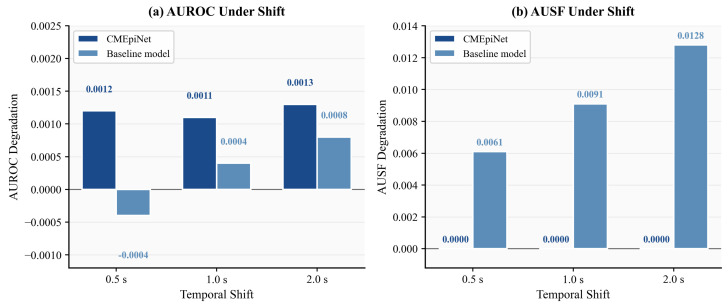
Performance degradation under auxiliary-modality temporal shifts.

Figure 10 shows the performance degradation of the two models relative to their own baselines under auxiliary-modality temporal shifts. In the temporal-shift experiments, EEG serves as the reference, and temporal offsets of 0.5 s, 1.0 s, and 2.0 s are applied to ECG and EMG. Figure 10a shows that both models maintain small AUROC degradation. Figure 10b shows that the AUSF degradation of CMEpiNet remains 0.0000 under all three temporal-shift conditions, whereas the AUSF degradation values of the baseline model are 0.0061, 0.0091, and 0.0128. These values increase gradually with the shift magnitude. This result indicates that CMEpiNet has stronger event-level stability against temporal asynchrony of auxiliary modalities.

This section analyzes model stability under perturbation conditions using AUROC and AUSF. AUROC reflects the window-level binary discriminative ability, whereas AUSF reflects the comprehensive trade-off between event-level detection performance and false alarm control under multiple thresholds. Therefore, these two metrics provide the main basis for robustness analysis.

In summary, CMEpiNet shows smaller relative performance degradation under window noise replacement and auxiliary-modality temporal shifts. It shows particularly stronger stability in terms of AUSF. This indicates that CMEpiNet has good robustness in the seizure detection task.

### 4.8. Computational Complexity Analysis

CMEpiNet remains compact in terms of model size, with 1.12 M parameters. It requires 12.3 GFLOPs to process a 60 s multimodal input, corresponding to 411 MFLOPs per 2 s window. On an RTX 4060 GPU, the average inference time is 0.17 ms per window under batch processing and 0.71 ms per window under single-window streaming, corresponding to real-time factors of $9\times 10^{-5}$ and $3.6\times 10^{-4}$, respectively. As summarized in Table 5, the real-time factor remains far below 1 in both settings, indicating that CMEpiNet supports faster-than-real-time inference. These results suggest that the proposed model remains computationally efficient despite its complex-valued multimodal design and has potential for long-term wearable seizure monitoring.

## 5. Conclusions

This study proposes a new multimodal seizure detection model, CMEpiNet, to achieve high-accuracy seizure detection using multisource physiological signals. The complex-valued multidomain feature extraction module extracts time-domain–phase features and time–frequency features from raw EEG, ECG, and EMG signals. This process provides a more comprehensive multiscale feature representation. The cross-modal alignment and fusion mechanism then reduces distribution differences among different modalities and strengthens seizure-related semantic consistency across modalities. This improves the quality of cross-modal feature fusion. Finally, the 3D spatial–modality attention classifier identifies the fused multimodal features and captures key dynamic seizure patterns from the time–feature–modality structure.

Experiments on the public SeizeIT2 multimodal epilepsy dataset show that CMEpiNet achieves better performance than baseline methods in seizure sensitivity and false alarm control. The ablation experiments verify the effectiveness of each core module. The robustness experiments further show that the model maintains stable detection performance under signal quality degradation and temporal asynchrony of auxiliary modalities.

The performance improvement of CMEpiNet mainly comes from three aspects. First, the complex-valued multidomain feature extraction module (CMFE) effectively enhances single-modality feature representation. Second, the cross-modal consistency constraint and latent-space alignment mechanism in DLSAF reduce structural differences among multimodal data. Third, the 3DCSA classifier efficiently captures multimodal dependencies in a three-dimensional space and improves the model’s ability to represent complex seizure-related dynamic structures.

This study has several limitations. First, all experiments were conducted on a single dataset, SeizeIT2. Although SeizeIT2 is a large-scale and clinically representative wearable epilepsy dataset, the cross-dataset generalizability of CMEpiNet has not yet been established on an independent external cohort. We used SeizeIT2 because it is one of the few public datasets that provide synchronized wearable EEG, ECG, and EMG recordings, which are required by the proposed multimodal framework. Commonly used public epilepsy datasets, such as CHB-MIT and EPILEPSIAE, do not provide this configuration. Evaluating CMEpiNet on these datasets would require removing the ECG and EMG branches, thereby reducing the task to single-modality EEG detection and preventing a like-for-like external validation of the proposed multimodal model.

Second, although the present evaluation includes overall and seizure-type-specific analyses, detailed per-patient performance analysis remains limited. The subject-level bootstrap confidence intervals partly quantify inter-patient variability, but they do not replace individualized evaluation. Future work should further explore personalized modeling and per-patient threshold adaptation to improve robustness across patients.

Overall, CMEpiNet shows promising event-level detection performance and robustness within the SeizeIT2 evaluation setting. However, the current FA/h rate should still be interpreted as a research-stage result rather than evidence of immediate readiness for clinical alarm deployment. Although CMEpiNet improves seizure-event sensitivity and achieves a better trade-off between sensitivity and false-alarm burden, false alarms remain an important limitation for long-term wearable monitoring. In addition, the proposed cross-modal alignment and three-dimensional attention modeling framework has good generality. It can be extended to other multimodal analysis scenarios, such as intelligent transportation and smart education, and provides a new technical pathway for complex event recognition.

## Figures and Tables

**Table 1:** Performance comparison with baseline methods on the SeizeIT2 test set. Sens, FA/h, and HMS are reported at a classification threshold of 0.5, whereas AUROC and AUSF are threshold-independent summary metrics.

Method	Sens (%)	FA/h	HMS	AUROC	AUSF
SVM [38]	71.10	11.00	66.70	0.6995	0.7229
ChronoNet [39]	84.20	100.50	44.00	0.7921	0.7953
InceptionTime [40]	58.60	9.18	54.93	0.6933	0.7566
CMEpiNet	86.44	21.41	77.88	0.6631	0.7611
95% CI	(75.49–94.77)	(17.88–25.35)	(66.92–86.29)	(0.6072–0.7125)	(0.6485–0.8577)

*Note:* Values in parentheses indicate 95% confidence intervals for CMEpiNet estimated by subject-level bootstrap resampling with 2000 resamples. Confidence intervals were not estimated for benchmark methods for which subject-level predictions were unavailable.

**Table 2:** Sensitivity of CMEpiNet by seizure type on the SeizeIT2 test set.

Seizure Type	TP	FN	Sens (%)
FBTC (focal to bilateral tonic–clonic)	55	0	100.00
FIA (focal impaired awareness)	101	19	84.17
FA (focal aware)	45	12	78.95
Focal (unspecified) ^*a*^	3	1	75.00
Overall	204	32	86.44

^*a*^ Only four events were available in this category; the result is listed for completeness and was not used for statistical inference.

**Table 3:** Ablation study results on the SeizeIT2 test set.

Model	Sens (%)	FA/h	AUROC	AUSF
Variant 1	52.15	7.26	0.6962	0.7597
Variant 2	68.04	9.46	0.6933	0.7786
Variant 3	80.26	14.13	0.6936	0.7620
CMEpiNet	86.44	21.41	0.6631	0.7611

**Table 4:** Performance degradation under different perturbation conditions.

Perturbation Condition	CMEpiNet		Baseline Model	
	AUROC	AUSF	AUROC	AUSF
20% window noise replacement	0.0019	0.0000	0.0068	0.0135
30% window noise replacement	0.0018	0.0000	0.0091	0.0314
0.5 s auxiliary-modality temporal shift	0.0012	0.0000	−0.0004	0.0061
1.0 s auxiliary-modality temporal shift	0.0011	0.0000	0.0004	0.0091
2.0 s auxiliary-modality temporal shift	0.0013	0.0000	0.0008	0.0128

**Table 5:** Computational complexity of CMEpiNet. RTF denotes the real-time factor (processing time divided by signal duration); a value below 1 indicates faster-than-real-time processing.

Metric	Value
Parameters	1.12 M
FLOPs (per 60 s input)	12.3 G
FLOPs (per 2 s window)	411 M
Inference time (batch, per window)	0.17 ms
Inference time (streaming, per window)	0.71 ms
Throughput (batch)	5771 windows/s
RTF (batch/streaming)	$9\times 10^{-5}$/$3.6\times 10^{-4}$

## Data Availability

The data presented in this study are openly available in OpenNeuro at https://doi.org/10.18112/openneuro.ds005873.v1.1.0, reference number ds005873.
